# Cross-Regional Cooperation and Counter-Market-Oriented Spatial Linkage: A Case Study of Collaborative Industrial Parks in the Yangtze River Delta Region

**DOI:** 10.3390/ijerph20021055

**Published:** 2023-01-06

**Authors:** Shaobo Wang, Junfeng Liu, Kunyao Xu, Meicheng Ji, Feifei Yan

**Affiliations:** 1 Institute of County Economic Development, Lanzhou University, Lanzhou 730000, China; 2 School of Business, Suzhou University of Science and Technology, Suzhou 215009, China; 3 School of Public Finance & Taxation, Shandong University of Finance and Economics, Jinan 250014, China; 4 School of Architecture and Urban Planning, Nanjing University, Nanjing 116029, China; 5 School of Economics and Management, Hunan Institute of Science and Technology, Yueyang 414006, China

**Keywords:** collaborative industrial parks, geographic expansion, complex network, driving mechanism, YRDR

## Abstract

At present, collaborative industrial parks (CIPs) in the Yangtze River Delta Region (YRDR) have become an important spatial strategy for coordinating regional development. However, existing studies tend to focus on individualized micro-studies, ignoring the regional-scale production space reconstruction by the geographical expansion of CIPs. Based on this, this study takes the YRDR, where the development of CIPs is relatively mature, as an example and systematically analyzes their geographic expansion process and driving mechanism. The results found that CIPs in the YRDR have gone through three stages: the exploration period of CIP construction under the guidance of assistance policies; the blowout development period of CIPs under the demonstration effect; and the complete cluster formation period of CIPs. Regional central cities, such as Suzhou, Hangzhou, Nanjing, and underdeveloped cities, such as Tongling and Xuancheng, are core nodes, with Shanghai–Nantong; Shanghai–Anqing; Nanjing–Huainan; Wuxi–Xuzhou; Suzhou–Suqian; and Jiaxing–Lishui being important elements in the flow channel. The CIP network is basically formed. During this period, the degree of all nodes increased to 134, the network connection rate increased to 2.26, and the network complexity was more significant. Furthermore, CIPs are essentially a form of capital re-territorialization and space restoration organized and coordinated by the government (provincial government or central government). In the meantime, the market and the social environment, such as residents’ living standards, urban development foundation, urban transportation, and urban investment, also have an important impact on the geographic expansion of CIPs. In the regression results, the coefficients of popu, finance, labor, and passenger are significantly negative, but the coefficients of wage, gdp, freight, and govrd are significantly positive.

## 1. Introduction

From the agricultural economy to the later stage of industrialization, the economy and society have basically been organized around cities [1]. However, in the era of knowledge economy, social and economic development needs have broadly expanded to regional areas [1]; economic production activities are constantly weakened by the rigid constraints of traditional administrative boundary [2]; and the attributes of cross-city and cross-regional production activities are becoming more and more obvious, with the production and living factors accelerating the flow and reconstruction at the regional scale, which finally triggers the transformation of the regional economic development model [3,4]. New economic development models, such as enclave economy and cross-border urban circle, which take CIP as an important spatial expression, are constantly emerging, and they have become an important means of reorganizing the regional economic system and the division of labor [5,6].

In the past, scholars discussed the spatial structure of economic and social connections between cities based on the flow of people, traffic, information, and capital [7,8]. The correlation characteristics of factor flows between cities are also the long-term focus of relational economic geography and evolutionary economic geography. Based on the correlation characteristics of factor flows, urban planners have designated a series of regional strategic plans and regional integrated development plans. However, in the multi-scale reconstruction of national space from urban entrepreneurialism to regionalization, CIP as a form of urban cooperation has rapidly developed in the YRDR and other regions and spread throughout the country, becoming an important space for coordinated regional development strategy [9]. Compared with the flow of people, traffic, information, capital, and other elements, CIP is more targeted in characterizing the production relationship between cities. At the same time, in the past, the spatial flow of factors mostly followed the geographical laws of proximity and agglomeration of wealthy clubs, while CIP under the intervention of the government broke the traditional geographical laws of proximity and agglomeration of wealthy clubs to a certain extent, making it possible to reconstruct regional production. In terms of economic connection structure, there are obviously different organizational models, and the spatial effect on regional economic development also presents obviously different characteristics.

With the development of CIPs in the YRDR, significant changes are taking place in the economic production links between cities. What changes have taken place in economic production linkages under the background of the expansion of CIPs in different places? How is their formation mechanism different from traditional element flow? It is an important direction for this paper to expand existing literature theory. As one of the typical representative regions of regional integrated development in China, the development of CIPs in the YRDR has experienced nearly 20 years. It has transformed from an exploration period to a gradual maturity period, which provides a good case support for us to carry out related research. Therefore, in order to clarify the heterogeneity of its connection with traditional factors for the reconstruction of economic production relations, this study takes the geographical expansion of CIPs in the YRDR as an example, and systematically analyzes the spatiotemporal evolution pattern of its geographical expansion based on complex network methods. At the same time, based on the two-way fixed effects measurement model, the mechanism of its geographical expansion is systematically discussed.

## 2. Literature Review

### 2.1. CIP and “Enclave Economy”

“Enclave Economy” first reflects the phenomenon that a foreign-oriented or high-tech industry is dominated by international capital rather than embedded in the local economy [5,6], which mainly reflects the multinational layout of the elements. At the international level, more attention is paid to regional cooperation across national borders, especially international cooperation within the EU [10,11,12]. Differences in national economic development levels, social and cultural environments, infrastructure construction levels, labor quality levels, governments’ governance levels, legal and regulatory systems, market environment construction, and economic operation management mechanisms and concepts all have an important impact on the multinational layout of parks [13,14].

Later, with the innovation of the interactive cooperation model between domestic cities, it is given new connotations, and the research scale begins to focus on regions or cities, which mainly reflects the cross-administrative boundary cooperation model between different cities or regions [15,16]. On the one hand, from the perspective of cities, enclave economy has become one of the important strategic means in the process of rapid urbanization [17]. Many cities guide the development and construction of outer suburban space by arranging industrial parks, new cities, and development zones. The enclave-style layout mode has accelerated the rapid expansion and spread of urban space for a long time. The cultivation of these strategic nodes also promotes the networked and multi-centralized development of cities. On the other hand, from a regional perspective, the networked layout of rapid transportation has led to the continuous emergence of cross-border cooperation models between different cities [18,19,20]. The cultivation of strategic space for CIPs has become an important platform for the exchange and cooperation of elements between different cities. Therefore, the construction of CIPs between different cities has become an important path to promote the development of regional economic integration. It should be noted that CIPs also occur within cities. However, in order to better analyze the evolution of the regional economic connection pattern under the networked layout of CIPs, this paper only analyzes the construction of CIPs in different cities.

### 2.2. Dynamic Mechanism of CIP

Current research mainly focuses on the economic performance [21], operation mode, classification, and formation mechanism of CIPs [22,23]. Some research studies focus on the cross-border flow of economic resources and discuss the function and performance of CIP in the allocation of regional resources [5]. Researchers have found that CIPs promote the cooperation in production, technology, capital, and other factors between different cities, especially in the process of co-construction, focusing on the development of industrial chains, which is conducive to the interaction and cooperation of production between cities. At the same time, some scholars also focus on the cross-border flow of governance power, discussing the power relationship and institutional arrangements of CIPs [24,25]. It is generally believed that a good benefit sharing mechanism and cooperation and a good exchange mechanism are the keys to the orderly development of CIPs. In addition, some scholars have also started with planning and management practices to summarize the mode of CIPs [16]. Among them, some researchers divide the enclave economy into two types: intra-regional cooperation and cross-regional cooperation. Other scholars also divide it into four modes: flying-into-the-land dominant mode, flying-out-of-the-land dominant mode, co-management mode by both parties, and multi-scale participation.

**Figure 1:**
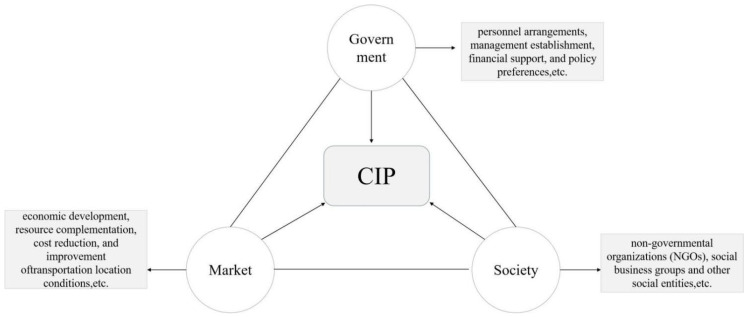
The dynamic mechanism of CIP.

As for the dynamic mechanism of CIPs (Figure 1), current discussion mainly centers on the government, the market, and the society. Among them, the government has an important influence on the practice of regional cooperation. The government drives the cooperation of the participants in CIPs through personnel arrangements, management establishment, financial support, and policy preferences [26,27,28], especially its policy support and intervention adopted by the local government based on the appeal of local interests, and it has become an important driving force and supercharger for CIPs to be initiated and continued cooperation [29]. In the meantime, some studies believe that CIPs have also become an institutional experiment to crack the administrative region’s economy: the selectivity of national space reflects the country’s preference for specific scales and fields, and this preference enables it to guide the reconstruction of national institutions and social economic space through CIPs [30,31].

The driving force of the market is the attractiveness of economic benefits brought about by the allocation of resources on a larger scale, and the effective flow of elements is gathered together to achieve the complementation and effective allocation of resources [10]. Some studies found that the economy of administrative regions has exacerbated the crisis of excessive capital accumulation, and severely restricted the improvement of regional competitiveness [32]. At the same time, due to differences in economic development, resource complementation, cost reduction, and improvement of transportation location conditions between regions, they have become important market driving forces for CIPs. The employment, income increase, and social welfare effects of the social entities brought about by the accumulation and allocation of market-driven elements promote the development of CIPs [33].

In addition, some studies also found that the public, non-governmental organizations (NGOs), social business groups, and other social entities play an important role in the development of CIPs. The cooperative partnership established by cooperative entities based on social factors, such as mutual trust, respect, and social network connections, plays an important role in promoting the transfer of knowledge in the process of cooperation [34]. NGO intervention has an important impact on park cooperation, especially for overseas CIPs. NGOs participate in local non-governmental activities, such as community organizations and agricultural or ecological projects, to promote the formulation of public policies, which in turn has an important indirect impact on the development of CIPs [35].

On the whole, current research mostly focuses on individualized micro-research, the scale is mainly concentrated in a city or even a CIP itself, and there is a lack of more detailed research on the changes and trends of macro-structure. In particular, it ignores the analysis of the restructuring of regional-scale production space by the geographical expansion of CIP. At the same time, current research on the driving mechanism of CIPs mainly focuses on government behaviors, and less attention is paid to the influence of the market and social environment. Based on this, the study takes the YRDR as example; conducts a systematic analysis of the spatial production process and organizational model evolution of CIPs; and adopts a combination of qualitative and quantitative methods to systematically summarize the impact of government behaviors, the market, and the social environment on CIPs, which can provide a theoretical reference for cross-urban regional organization of economic activities in the new era.

## 3. Methodology

### 3.1. Two-Way Fixed Effects Model

Considering the availability of data, this article adopts a relatively qualitative analytical paradigm when analyzing government influence, and a quantitative analytical paradigm when it comes to market and social influence. Based on the literature review, this article further explores the influence of market and social factors on CIPs with a two-way fixed effects measurement model [36,37,38,39]:

(1) $$K_{it}=\alpha_{0}+\sum^{\;}\beta_{i}X_{i}+city_{i}+year_{i}+\varepsilon_{it}\;$$

where *i* represents the individual city; *t* represents the year; *K* represents the degree value of the dependent variable node (considering that the degree value of a city can better reflect the degree of the city’s participation in CIPs, this study selects the degree value of city as the dependent variable); *X* represents the driving factor variable; city is the unobservable individual fixed effect of a city; *year* is the time fixed effect; and *ɛ* is the random disturbance term. With reference to previous research literature [15,16,17,21,22,33], based on the availability of city data, a specific driving factor variable index system is constructed (Table 1).

① Residents’ living standards. This variable mainly includes the degree of population agglomeration, wage level, and housing price level. This analysis assumes that the living standards of residents reflect the production and operation costs of different cities (labor costs, land price costs, etc.), and cost differences are one of the important considerations for the expansion of factors.

② The foundation of urban development. This variable mainly includes economic development level, financial development level, and industrial structure. This analysis assumes that the foundation of urban development has an important influence on the attraction of external elements.

③ Urban traffic conditions, mainly including highway passenger traffic, highway freight traffic, and the number of taxis. This analysis assumes that urban traffic conditions reflect the convenience of inter-city transportation, which will have an important impact on the cooperation and economic relations between cities.

④ City’s investment intensity, mainly including the number of employees, the level of human capital, and government scientific research support. This analysis assumes that the intensity of urban investment represents the construction capacity of CIPs.

It is necessary to explain that, in order to obtain the degree of a city’s participation in CIPs and systematically analyze the temporal and spatial evolution characteristics of CIPs, this study firstly constructs a CIP network and then uses a complex network analytical method to systematically analyze the characteristics of the CIP network.

In terms of data, as for the dependent variable Degree, this paper calculates the Degree of 40 cities in the Yangtze River Delta from 2003 to 2019 according to the number of CIPs by any two nodes. As for the independent variables, since the sample of this paper is the data of 40 cities in the Yangtze River Delta from 2003 to 2019, the data of relevant indicators are from the Statistical Yearbook of Chinese Cities, and the housing price data come from the Macroeconomic and Real Estate Database of the National Information Center.

### 3.2. Complex Network Analytical Method

CIPs can reflect the economic interaction between two cities. This research takes cities as the network node, takes the existence of CIPs between two cities as the edge, and uses the number of CIPs between the two cities as the connection strength to construct a complex network model H [40,41,42,43].

(2) $$H=\left(V,E,W,W^{\prime },t\right)\;$$

where *V* represents the set of all nodes; *E* is the set of all edges (the connection of CIPs between two cities); *W* and *W′* represent the attributes of all the nodes and all the edges; and *t* is the set of network years.

Degree: It mainly reflects the number of direct connections between network nodes and other nodes [44]. Degree can reflect the importance of a node in the network. In general, the larger the node degree, the stronger its influence in the network. The specific formula is as follows:

(3) $$K=\overset{N\left(t\right)}{\underset{j=1}{\sum }}a_{ij}\;$$

where *K* represents the degree value of each node; *a_ij_* represents the construction of CIPs between city *i* and city *j*; and *N*(*t*) represents the total number of network nodes.

Connection rate: It mainly reflects the complexity of the network. When the connection rate is 0, it means that the network is a completely disconnected network, which shows a scattered point distribution. When the connection rate is less than 1, it means that the network is in a tree structure or there are discrete sub-networks; when the connection rate is greater than 1, it means that the network has a loop structure. Generally speaking, the greater the connection rate, the more complex the network.

(4) l=en 

where *e* represents the actual number of CIPs, and *n* represents the number of nodes in the network.

Network density: It refers to the ratio of the actual number of CIPs to the total number of possible CIPs, and it is used to measure the overall tightness of the network. The larger the value, the higher the tightness of the network. On the contrary, a smaller value means sparser network connection. The density of the network ranges from [0, 1].

(5) d=2en(n−1) 

Centrality: In recent years, in order to fully evaluate the status and role of nodes in the network, scholars have mainly analyzed the degree centrality, adjacency centrality, and betweenness centrality [43].

Degree centrality: Degree reflects the connection between a node and other nodes. Degree centrality is the most direct measure to describe the centrality of nodes. The greater the degree value of nodes, the higher the degree centrality of these nodes and the greater the importance of these nodes in the network [41]. The specific calculation formula is as follows:

(6) Ki′=∑i=1naij(n−1) 

### 3.3. Study Area and Data Sources

**Figure 2:**
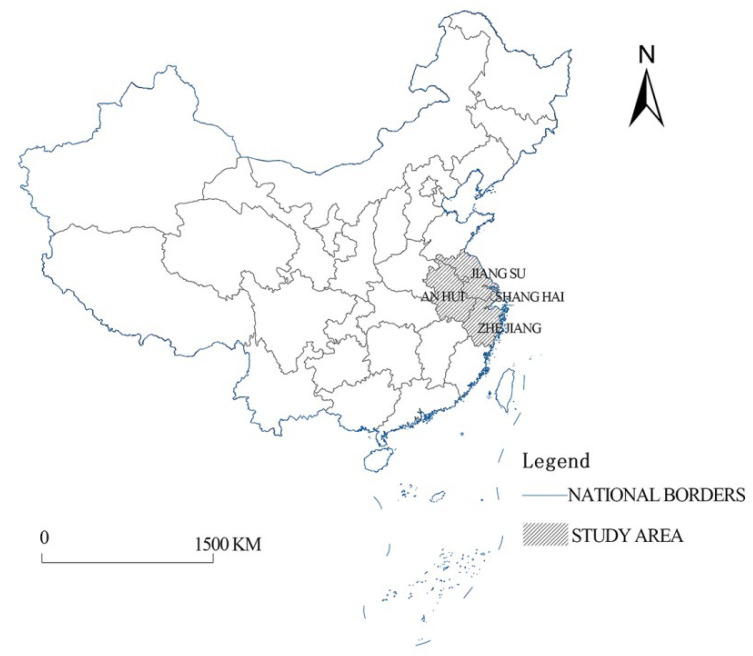
Research area.

This study takes the Yangtze River Delta Region as the research area (Figure 2). According to the 2019 version of the Outline of the Yangtze River Delta Regional Integration Development Plan, the Yangtze River Delta Region involves four provinces (cities), including Shanghai, Jiangsu, Zhejiang, and Anhui, and 41 cities in the region. There are two main reasons that we choose the Yangtze River Delta as the research region: on the one hand, the Yangtze River Delta is one of the regions with the highest degree of regional integration development in China, which has important reference significance for guiding the integration development of other regions. On the other hand, in China, the construction of CIP in the Yangtze River Delta started at the earliest stage and is now relatively mature, which can provide a typical case for this study.

Taking into account that relevant data on CIPs have not been systematically counted, the research process mainly uses policy documents and the literature to sort out the data, while taking into account the information extracted from news materials and reports to obtain relevant data. Secondly, part of the data is obtained in conjunction with the statistical yearbooks of the development zones of various provinces and cities. These statistics found that after 2010, CIPs in the Yangtze River Delta entered a period of blowout development. At present, according to incomplete statistics (Table 2), the number of CIPs has reached 153. The number of cross-provincial CIPs, cross-city CIPs within the province, cross-district(county) CIPs within the province, and government-enterprise CIPs is 26, 88, 10, 29, respectively. Among them, most of the parks are cross-city CIPs within the province, and the three provinces of Jiangsu, Zhejiang, and Anhui currently have more than 40. Among the cross-provincial CIPs, Shanghai has led the most pairings with 19, mainly located in the Jiangsu and Anhui provinces. Secondly, Jiangsu and Anhui, and Zhejiang and Anhui jointly built 4 and 3 CIPs, respectively. On the whole, CIPs are currently densely distributed in the Yangtze River Delta, and have gradually developed into an important driving force for the reconstruction of the Yangtze River Delta’s production space. It should be noted that this research mainly discusses the role of CIPs in the restructuring of regional production space. It is mainly based on the complex network of two cities with CIPs. Therefore, this study does not consider cross-district(county) CIPs within the province and government-enterprise CIPs.

As for the data in Table 1,the unbalanced panel data from 2003 to 2019 mainly come from the “China City Statistical Yearbook”, and the housing price data come from the Macroeconomic and Real Estate Database of the National Information Center.

## 4. Results

### 4.1. Spatiotemporal Distribution Characteristics of CIP

Overall, the geographical expansion of CIPs in the YRDR can be roughly divided into three stages: the exploration period of CIPs; the “blowout” development period of CIPs; and the complete cluster formation period of CIPs. In different stages, there are obvious differences in the spatial organization mode of CIPs.

#### 4.1.1. Exploration Period of CIPs

The establishment of a Jiangyin–Jingjiang CIP in 2003 opened the prelude to CIPs in the YRDR. In 2006, in order to solve the backward economic development in the northern part of Jiangsu, the Jiangsu Provincial Government issued the “North-South Linkage” policy, which has further explored the institutional design of CIPs under the leadership of the government [44,45]. Under administrative instructions, cities in the southern part of Jiangsu, including Suzhou, Wuxi, Changzhou, and Nanjing, began to carry out counterpart exchanges and cooperation with the cities in the northern part of Jiangsu, and started the construction of cross-city CIPs in Suqian, Lianyungang, Xuzhou, Yancheng, Huai’an, and other places in the northern part of Jiangsu. As of 2010, Jiangsu had built 19 North–South linkage CIPs. During this period, the number of CIPs built by Suzhou and Suqian and by Wuxi and Xuzhou is five and six, respectively. At the same time, under the influence of Jiangsu’s system exploration, Shanghai and other places had gradually begun the process of CIP construction.

**Figure 3:**
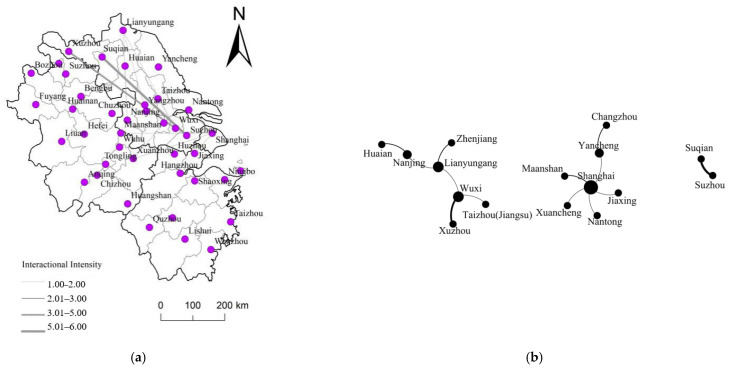
(**a**) is the spatial connection pattern during the exploration period, and (**b**) is the spatial organization mode of CIPs during the exploration period.

At this stage, CIPs basically present a one-way connection, and under the influence of assistance policies, CIPs are basically set up in underdeveloped cities (Figure 3a). In order to better present the spatial organization mode of these CIPs, this research is based on the Gephi platform to construct the network organization structure of these CIPs (Figure 3b). It is found that the connection network forms three relatively independent network organization clusters as a whole, with Shanghai, Suzhou, and Wuxi as the core hub nodes, respectively guiding the economic activity cooperation within a certain range. At the same time, Suzhou and Suqian, and Wuxi and Xuzhou have the strongest links to each other, forming a connecting axis between the two clusters. In addition, during this period, Shanghai presents a finger-like spatial organization mode with its super radiation ability. On the whole, there are few connections between the network nodes during this period, and the network presents a simple “spindle-spoke” organization model.

From the characteristics of the complex network at this stage, there are 11 nodes with a degree of one, accounting for 68.75%. From the perspective of degree centrality, Shanghai has the highest degree value (5) and has the most connections with other nodes in the network, showing high centrality (0.33). This is followed by Lianyungang, Yancheng, Wuxi, and Nanjing (degree value 2), with the degree centrality being all 0.13. In terms of the connection rate and network density values, both are zero, indicating that the interaction and interdependence between these node cities are relatively limited, and the network structure is relatively simple at this time.

#### 4.1.2. “Blowout” Development Period of CIPs

Inspired by the CIPs in Jiangsu, Zhejiang carried out the construction of the “Mountain-Sea cooperation” CIPs, and Anhui carried out the “Southern and Northern cooperation” CIPs. Zhejiang issued the “Several Opinions on the Implementation of a New Round of Mountain-Sea CIP Projects” in 2009 [46], encouraging developed regions to help underdeveloped regions in pairs.

**Figure 4:**
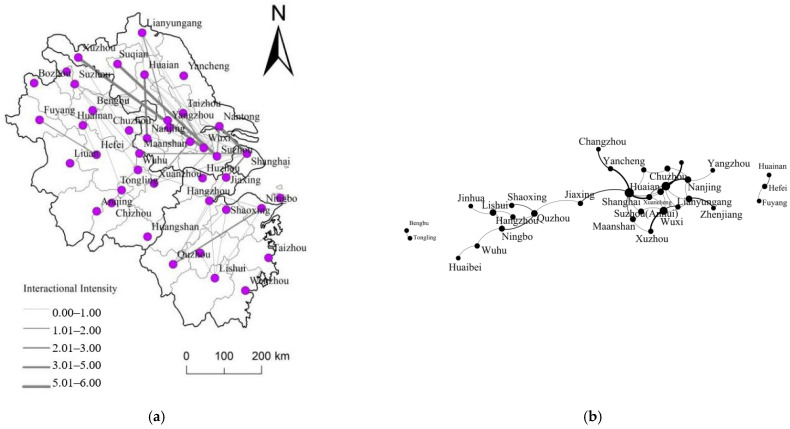
(**a**) is the spatial connection pattern during the “Blowout” development period, and (**b**) is the spatial organization mode of CIP during the “Blowout” development period.

At this stage, the scale of CIPs had rapidly expanded and had expanded to Zhejiang and Anhui provinces (Figure 4a). The number of nodes participating in the construction of CIPs increased from 16 to 32, an increase of 100%. From the perspective of the spatial organization model (Figure 4b), independent organizations with Shanghai, Suzhou, and Wuxi as the core hub nodes are connected as a whole due to the continuous expansion of the branch network. The network nodes between Shanghai and Jiangsu are directly connected, and the integration process is highlighted. At the same time, for Zhejiang Province, the integration of Jiangsu, Zhejiang, and Shanghai has entered a new stage through Jiaxing. However, for Anhui Province, there is still a certain separation from the Jiangsu, Zhejiang, and Shanghai network during this period. Bengbu–Tongling and Fuyang–Hefei–Huinan organization clusters are independent of the network, and still present three relatively independent organization clusters in space.

From the characteristics of the complex network at this stage, the degree value of all nodes has increased from 24 to 83, an increase of 245%. At the same time, node cities have begun to show a multi-directional development pattern. Among them, there are 13 node cities with a degree value of more than three, accounting for 40.6%. Especially for Shanghai, Suzhou, Wuxi, Nanjing, which have relatively good economic foundations, with degrees of 8, 7, 6, and 4, respectively. These node cities also show high centrality in the network of CIP, with high agglomeration coefficient and node centrality, which makes the hub-and-spoke network structure characteristics outstanding during this period. At the same time, during this period, the network connection rate has increased from 0.85 to 1.59, and the network complexity has increased significantly. The network density has dropped from 0.20 to 0.17, and the overall tightness of the network has dropped. From the distribution of CIP in various provinces, the scale of CIPs in Jiangsu Province continues to expand, especially between Nanjing and Huai’an, between Changzhou and Yancheng, and between Lianyungang and Zhenjiang, and the number of CIP (three or more) has increased significantly. In Anhui Province and Zhejiang Province, CIPs have begun to appear sporadically, but the number of CIPs between urban nodes is basically one; the network connection rate and network density are low, and the network structure is relatively simple. As far as Shanghai is concerned, Shanghai has started cross-provincial CIPs, with Nantong, Yancheng, and Suzhou in Jiangsu Province; Xuancheng and Ma’anshan in Anhui Province; and Jiaxing in Zhejiang Province. However, during this period, CIPs in the YRDR are basically within the province and the newly built CIPs are mainly concentrated in Jiangsu and Anhui provinces, while the number of newly built CIPs in Zhejiang is still relatively small.

#### 4.1.3. The Complete Cluster Formation Period of CIPs

After 2015, the National Development and Reform Commission of China issued the “Guiding Opinions on Supporting the Development of “Enclave Economy”” [9]. Under the guidance of national policies, the enclave economy in the YRDR has entered a new round of development. On the one hand, the government-led model has changed, and government-enterprise cooperation has become a new means of CIP construction. On the other hand, the mode of cross-city CIPs within the province has changed significantly, and the number of cross-provincial CIPs has increased significantly.

**Figure 5:**
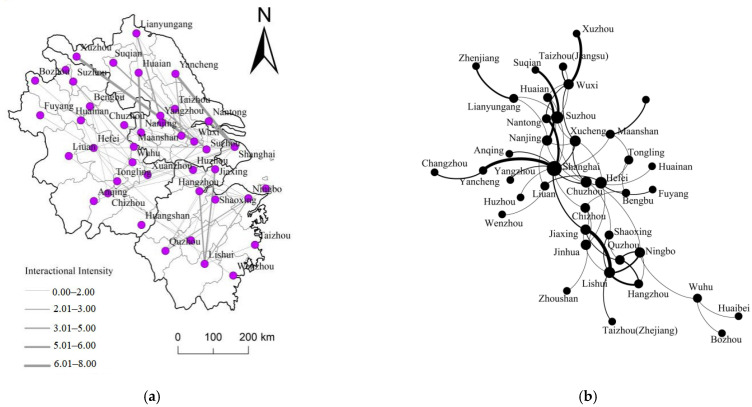
(**a**) is the spatial connection pattern during the formation period of a complete cluster, and (**b**) is the spatial organization mode of CIPs during the formation period of a complete cluster.

At this stage, the scale of CIPs continues to expand, and with the development of cross-provincial CIPs, the interconnection of elements between cities has basically been realized (Figure 5a). From the perspective of spatial organization mode (Figure 5b). the CIP of Jiangsu, Zhejiang, Shanghai, and Anhui will be directly connected, and the integration process will be significantly improved. So far, the network of CIPs has formed a multi-core hub-and-spoke structure with Shanghai as the core and Suzhou, Ningbo, Hefei, and Nanjing as the secondary cores. At the same time, from the network connection axis, Shanghai–Nantong, Shanghai–Anqing, Nanjing–Huainan, Wuxi–Xuzhou, Suzhou–Suqian, and Jiaxing–Lishui have the strongest connections to each other, and they have also become the main channels for the regional flow of economic factors in the YRDR.

From the characteristics of the complex network at this stage, the number of nodes participating in CIPs has increased from 32 to 41, and the degree of all nodes has increased from 83 to 134. During this period, the network connection rate has increased from 1.59 to 2.26, the network complexity has become more significant, and the network has a loop structure. The network density has dropped from 0.17 to 0.16, indicating that, with the geographical expansion of CIPs, the overall network compactness has declined, and the overall network connection has become relatively sparse, which is closely related to the connection between some new nodes in Anhui and Zhejiang. From the perspective of the degree value of the network nodes, there are 11 nodes with a degree value of one in the network, accounting for 26.2%. There are 22 node cities with a degree value of more than three, accounting for 53.7%. The nodes with a degree value of more than five are Shanghai, Suzhou, Hefei, Xuancheng, Ningbo, Lishui, Chuzhou, Wuxi, and Nanjing. Among them, Shanghai, Suzhou, Hefei, Ningbo, Wuxi, and Nanjing are mainly flying-out places, while Xuancheng, Lishui, and Chuzhou are mainly flying-into places. From the perspective of agglomeration coefficient and centrality index, the formation of complete agglomeration further strengthens the network centrality characteristics of many nodes. On the one hand, some cities with high degree have formed a high network centrality, such as Shanghai, Hangzhou, Suzhou, and Nanjing. On the other hand, some cities with relatively backward development foundations are also closely connected with other nodes in the network, such as Tongling and Xuancheng, which also exhibit high network centrality characteristics.

To sum up, with the development of cross-provincial CIPs, the complete cluster formation of the network has become more and more obvious, and it has become an important regional space carrier that carries the occurrence and development of economic activities in the YRDR. The spatial distribution of CIP plays an important role in promoting the process of industrial integration in the YRDR.

### 4.2. Driving Mechanism of the Geographical Expansion of CIPs

Based on previous research, it is not difficult to find that the evolution of CIPs will not only be influenced by policy guidance, but will also be affected by the market, social, and other factors. It should be noted that in China, social organizations are relatively limited in guiding the construction of CIPs, so this paper does not discuss the social aspects.

#### 4.2.1. Impact of Local System Experiment and National Spatial Choice on the Geographic Expansion of CIPs

As a new institutional space, a CIP is fundamentally driven by the spatial movement of capital [47], and it is also a part of local and national institutional experiments [9,25]. Since the reform and opening up, in some of the developed central cities in the YRDR, capital has become saturated due to excessive accumulation (in the form of repeated construction of infrastructure, etc.), factors such as land and labor have become increasingly tense, and the phenomenon of agglomeration has become increasingly prominent. This seriously restricts urban development. The administrative regional economy has aggravated the crisis of excessive capital accumulation in the city itself, and has also led to the widening of the development gap in the YRDR, which has severely restricted the improvement of regional competitiveness. In this context, CIP has also become an institutional experiment to crack the economics of administrative regions [21]. As a system experiment, CIPs were mainly manifested in the policy support in the province for the purpose of helping each other in the initial stage of exploration. Jiangsu, Zhejiang, Anhui, and others successively introduced relevant assistance policies to support CIPs. During this period, CIPs directly relied on the counterpart assistance between cities to develop. The space was mainly short-distance connections, and cities with close geographical relations or long-term cooperative relations were selected.

In addition, the national spatial selectivity has played a significant role in promoting the development of cross-provincial CIPs [24]. It is mainly reflected in the country’s preference for specific scales and fields, and this preference enables it to guide the reconstruction of national institutions and socio-economic space through national space projects or strategies [48]. Especially after the National Development and Reform Commission issued the “Guiding Opinions on Supporting the Development of Enclave Economy” in 2015, CIPs have risen from a bottom-up system experiment to a national spatial strategy, and have superimposed on original strategies, such as urban agglomeration planning, and they have become a new means of restructuring national space. The “YRDR City Group Development Plan” has clearly stated that the YRDR City Group is an important platform for China to participate in international competition. The enclave economy represented by CIPs not only provides space for industrial transformation and upgrading of central cities, but also enables peripheral cities to obtain development factors. Therefore, it plays an important role in promoting regional integration and improving the overall competitiveness of the region. In this context, the development of cross-provincial CIPs is accelerating, and the phenomenon of organized leapfrogging cross-provincial enterprise relocation based on the economic complementarity between cities has become more and more obvious, which can realize the re-territorialization of capital space and guide the integrated development of factors. As a whole, CIPs are essentially a form of capital re-territorialization and space restoration coordinated and organized by the government [49].

#### 4.2.2. Impact of the Market, Social, and other Factors on the Geographic Expansion of CIP

In order to reflect the robustness of the regression results regarding the driving mechanism, this paper conducts a stepwise regression test according to the four variable groups involved. The results demonstrate that the influence of different market factors on CIPs shows obvious heterogeneity (Table 3). For the sake of avoiding the error of regression results caused by the multiple collinearity between independent variables, based on the VIF test and Pearson correlation coefficient test, this paper finds that the multiple collinearity of variables has passed the test, that is, the variable selection is reasonable and the model estimation result is reliable.

On the whole, affected by the strong government intervention, the geographical expansion of CIPs has broken the cognitive category of traditional economic geography that the flow of factors follows geographical proximity and agglomeration to the megacities; the impact of some factors on the geographical expansion of CIPs presents an anti-marketization characterization. In the regression results, the coefficients of popu, finance, labor, and passenger are significantly negative, indicating that the higher the level of urban population agglomeration and financial development; the more the number of people; and the greater the passenger traffic, the lower the enthusiasm for participating in the construction of CIPs. This phenomenon is contrary to the laws of the market, which may have a lot to do with the development stage of most cities with a high level of economic foundation development in the YRDR. At present, most cities in the YRDR with high development level cannot meet their own development needs in terms of population absorption and financial services, etc. Under the influence of government support policies, they have to participate in the development of CIPs, which makes some factors appear to have anti-market characterization.

At the same time, the coefficients of wage, gdp, freight, and govrd are significantly positive. It shows that the geographical expansion of CIPs is still positively affected by market factors, such as the level of local economic development, external production links, the level of scientific and technological development, and the income of residents. This analysis assumes that, with the market-oriented development of CIPs, the foundation of economic development, the connection with external production, the level of scientific and technological development, and the income of residents have become the key factors affecting the high-quality and sustainable development of CIPs, and they have also become an important manifestation of market participation in the construction of CIPs.

In addition, the coefficient of house is positive but not significant; the coefficient of sec is negative but not significant; and the coefficient of hum is negative but not significant.

#### 4.2.3. Robustness Tests

In order to ensure the reliability of the regression results of the above-mentioned driving mechanism, this paper also conducts a series of robustness tests.

① Stepwise regression. The stepwise regression results in Table 4 show that the size and significance of the regression coefficients of each driving variable remain relatively stable, indicating that the model estimation results have a certain degree of robustness.

② Change the research sample. Table 4 Model (2) is the regression results with Shanghai and Anhui as samples, and Table 4 Model (3) is the regression results with Shanghai, Jiangsu, and Zhejiang as samples. Compared to the benchmark regression results of Table 4 Model (1), each driving variable also remains relatively stable.

③ Select some years. Model (4) in Table 4 is the regression results of the research samples before 2015, and Model (5) in Table 4 is the regression results before 2016. Compared to the benchmark regression, it is found that the overall regression level has not changed significantly, and the results are still have robustness.

## 5. Conclusions and Discussions

### 5.1. Conclusions

In recent years, the policies and practices of CIPs in Jiangsu, Anhui, Zhejiang, Shanghai, and other places have continued to innovate. CIPs have shown the characteristics of diversified types, multiple participants, and cross-regional cooperation. This article conducts empirical research on the evolution and driving mechanism of CIPs in the YRDR. The results demonstrate the following:

From the perspective of the geographical expansion of CIPs, we found that, with the geographical expansion of CIPs, city nodes are gradually connected due to the co-construction relationship, and a networked organization model of CIPs has emerged. At present, the core nodes of regional central cities, such as Shanghai, Suzhou, Hangzhou and Nanjing, and underdeveloped cities, such as Tongling and Xuancheng, have been initially formed, with Shanghai–Nantong; Shanghai–Anqing; Nanjing–Huainan; Wuxi–Xuzhou; Suzhou–Suqian; Jiaxing–Lishui, and others being the important elements in the flow channel. The construction of CIPs under the assistance policy has boosted the central position of urban nodes in some underdeveloped regions to a certain extent, and will have a greater impact on the development of the edge cities of the YRDR.

From the perspective of the driving mechanism of CIPs, on the one hand, CIPs are a form of the capital re-territorialization and space restoration coordinated and organized by the government (provincial government and central government). On the other hand, affected by the strong government intervention, the impact of some factors on the geographical expansion of CIPs present anti-market characterization, with the coefficients of popu, finance, labor, and passenger being significantly negative. However, market factors, such as the local economic development level, external production connection, scientific and technological development level, and residents’ income, still play a significant positive role in the geographical expansion of CIPs.

In this context, we should give full play to the important role of CIPs in the cross-regional flow of factors, actively guiding developed cities and surrounding underdeveloped cities to carry out the construction of CIPs, guiding cross-regional exchanges and cooperation of factors, and promoting a new round of development of regional integration. Secondly, we should give full play to the dual factors of government and market in future urban and regional development. On the one hand, we should give full play to the government’s macro-control role in guiding the cross-regional flow and connection of elements, giving more development opportunities to marginal cities or underdeveloped areas, and promoting the rapid development of integration by creating policy space, such as CIPs in different places. On the other hand, we should also provide a good market environment for the cross-regional flow of factors, such as the construction of rapid transit system, supporting urban infrastructure, giving full play to the guiding role of the market, and removing the market barriers of cross-regional circulation of factors.

### 5.2. Discussions

This research systematically sorts out the process and driving factors of the geographical expansion of CIPs in the YRDR, which can provide a certain theoretical reference for the production interaction in the YRDR in the new period. On the one hand, in the past, scholars discussed the spatial structure of economic and social connections between cities based on the flow of people, traffic, information, and capital [7,8], and the spatial flow of factors mostly followed the geographical laws of proximity and agglomeration of wealthy clubs. This paper further verifies and summarizes the anti-marketization spatial layout behavior of the expansion of CIPs under the leadership of the government. Affected by the strong government intervention, the geographical expansion of CIPs has broken the cognitive category of traditional economic geography that the flow of factors follows the geographical proximity and the agglomeration to the megacities. The impact of some factors on the geographical expansion of CIPs present anti-marketization characterization. More surrounding cities have more development opportunities, which is of great practical significance for promoting the development of the whole region and integrated development. On the other hand, this study breaks previous research assumption regarding CIPs by focusing on institutional arrangements and points out that the construction of CIPs in different places will also be affected by some market factors, such as the local economic development level, external production connection, scientific and technological development level, and residents’ income.

However, there are still many problems that need to be further analyzed and improved. On the one hand, this research lacks relatively micro-data, such as the industrial attributes of parks, and the analysis of the spatial impact of CIPs on the industrial structure of the YRDR is lacking. On the other hand, current parks have experienced nearly 20 years of development. How are their development status? What are the later issues that they are facing? Furthermore, this paper only analyzes the process and driving factors of the geographical expansion of CIPs, and there is no validation on whether CIPs play a mediating role in the cross-regional flow. All of these questions need to be analyzed through a field survey system.

## Figures and Tables

**Table 1:** Driving mechanism for the geographical expansion of CIPs.

Variable	Variable Group	Variable Name	Symbol	Index Calculation
Dependent variable	Degree	Degree	*K*	$K=\sum_{j=1}^{N(t)}aij(t)$
Independent variable	Residents’ living standards	The degree of population agglomeration	*popu*	Total population at the end of the year/land area of administrative area
		Wage level	*wage*	Log value of total wages of employees
		Housing price level	*house*	Average price of urban housing
	The foundation of urban development	Economic development level	*gdp*	Log value of GDP
		Financial development level	*finance*	Log value of the balance of loans of financial institutions at the end of the year as a percentage of GDP
		Industrial structure	*sec*	The added value of the secondary industry as a percentage of GDP
	Urban traffic conditions	Highway passenger traffic	*passenger*	The ratio of highway passenger traffic to the total population at the end of the year
		Highway freight traffic	*freight*	The ratio of road freight volume to total population at the end of the year
		The number of taxis	*taxi*	The log value of taxis
	City’s investment intensity	The number of employees	*labor*	Log value of the number of employees in urban units at the end of the period
		The level of human capital	*hum*	Log value of the ratio of the number of ordinary undergraduates and junior college students to the total population at the end of the year
		Government scientific research support	*govrd*	Log value of the ratio of science and technology expenditure to budget expenditure

**Table 2:** Statistics of types of jointly constructed parks.

Region	Type	Number	Region	Type	Number
Jiangsu	cross-city within the province	38	Anhui	cross-city within the province	13
	government-enterprise	6		cross-district (county)	2
	cross-provincial (Shanghai–Jiangsu)	12		government-enterprise	23
Zhejiang	cross-city within the province	37		cross-provincial (Shanghai–Anhui)	6
	cross-district (county)	8		cross-provincial (Zhejiang–Anhui)	4
	cross-provincial (Shanghai–Zhejiang)	1		cross-provincial (Jiangsu–Anhui)	3

**Table 3:** Measurement test of the driving mechanism.

Model	(1)	(2)	(3)	(4)
Variable	*K*	*K*	*K*	*K*
*popu*	−0.6714 **	−0.6301 **	−0.9727 ***	−0.9569 ***
	(0.2443)	(0.2491)	(0.2571)	(0.2550)
*wage*	1.2278 ***	0.9874 **	0.6301 *	1.2390**
	(0.1640)	(0.3357)	(0.3390)	(0.3881)
*house*	0.1830	0.2314	0.2107	0.4132
	(0.3576)	(0.4136)	(0.4274)	(0.5114)
*gdp*		0.2904	0.6837 *	0.8681 **
		(0.3677)	(0.3682)	(0.3815)
*finance*		−0.7543 *	−0.7415	−1.0661 *
		(0.4554)	(0.4896)	(0.5908)
*sec*		−1.6543 **	−0.3749	−0.6159
		(0.7175)	(0.8064)	(0.8751)
*passenger*			−0.6910 ***	−0.6716 ***
			(0.1541)	(0.1696)
*freight*			0.6912 ***	0.7158 ***
			(0.2014)	(0.2092)
*taxi*			0.2236	0.3458 *
			(0.1747)	(0.1964)
*labor*				−0.9417 **
				(0.3452)
*hum*				−0.0157
				(0.1689)
*govrd*				0.2837 **
				(0.1143)
*_cons*	−19.2823 ***	−14.3891 **	−23.4813 ***	−33.2232 ***
	(2.2094)	(4.5401)	(4.5417)	(5.9435)
*N*	399	359	359	359
*R-sq*	0.310	0.327	0.402	0.421

Note: *, **, *** indicate significance at the statistical level of 10%, 5%, and 1%, respectively; the value in () indicates the robust standard deviation of the regression coefficient.

**Table 4:** Robustness test of the driving mechanism.

Model	(1)	(2)	(3)	(4)	(5)
Variable	*K*	*K*	*K*	*K*	*K*
*popu*	−0.9569 ***	−1.4877 ***	−0.2878	−0.6499 **	−0.5929 **
	(0.2550)	(0.4325)	(0.4054)	(0.2975)	(0.2715)
*wage*	1.2390 **	2.2326 ***	1.1743 *	1.6065 **	1.3587 **
	(0.3881)	(0.6340)	(0.6299)	(0.4978)	(0.4473)
*house*	0.4132	3.4795 ***	0.1677	0.0207	0.0319
	(0.5114)	(0.8828)	(0.7339)	(0.5793)	(0.5372)
*gdp*	0.8681 **	−0.7939	2.3439 ***	1.0948 **	1.1009 **
	(0.3815)	(0.7086)	(0.6753)	(0.3891)	(0.3727)
*finance*	−1.0661 *	−2.5042 **	−0.1041	−1.7092 **	−1.5459 **
	(0.5908)	(0.8347)	(0.8787)	(0.7244)	(0.6602)
*sec*	−0.6159	−0.7353	−3.5218 **	−1.4920	−1.3062
	(0.8751)	(1.2501)	(1.5517)	(0.9894)	(0.9284)
*passenger*	−0.6716 ***	−0.6221 **	−0.5131 **	−0.5169 **	−0.4429 **
	(0.1696)	(0.2831)	(0.2350)	(0.2068)	(0.1941)
*freight*	0.7158 ***	0.4132	0.5362 *	0.8097 **	0.7584 ***
	(0.2092)	(0.3711)	(0.3175)	(0.2528)	(0.2270)
*taxi*	0.3458 *	0.3739	−0.1325	0.0958	0.1216
	(0.1964)	(0.5938)	(0.3194)	(0.2301)	(0.2085)
*labor*	−0.9417 **	−0.4766	−1.8711 **	−1.3792 **	−1.1749 **
	(0.3452)	(0.3688)	(0.6654)	(0.4488)	(0.3885)
*hum*	−0.0157	−0.1086	−0.1042	−0.0637	−0.0938
	(0.1689)	(0.2520)	(0.2628)	(0.2388)	(0.2197)
*govrd*	0.2837 **	0.4983 ***	0.5531 **	0.0378	0.0004
	(0.1143)	(0.1456)	(0.1792)	(0.2864)	(0.2681)
*_cons*	−33.2232 ***	−47.0013 ***	−34.5649 ***	−31.5124 ***	−29.7705 ***
	(5.9435)	(8.0575)	(10.0838)	(7.1302)	(6.4714)
*N*	359	144	224	200	240
*R-sq*	0.421	0.730	0.436	0.435	0.417

Note: *, **, *** indicate significance at the statistical level of 10%, 5%, and 1% respectively; the value in () indicates the robust standard deviation of the regression coefficient.

## Data Availability

Data are available from the authors upon request.

## References

[1] Chen C.L., Peter H. (2011). The impacts of high-speed trains on British economic geography: A study of the UK’s intercity 125/225 and its effects. J. Transp. Geogr..

[2] Yang C. (2005). Multilevel governance in the cross-boundary region of Hong Kong Pearl River Delta, China. Environ. Plan. A.

[3] Luo X., Shen J. (2009). A Study on inter-city cooperation in the YRDR, China. Habitat Int..

[4] Shen J. (2014). Not quite a twin city: Cross-boundary integration in Hong Kong and Shenzhen. Habitat Int..

[5] Li Y., Wu F. (2012). Towards new regionalism? Case study of changing regional governance in the Yangtze River Delta Region. Asia Pac. Viewp..

[6] Li Y., Wu F. (2018). Understanding city-regionalism in China: Regional cooperation in the YRDR. Reg. Stud..

[7] Shang H., Jiang L., Pan X.F. (2022). Does R&D element flow promote the spatial convergence of regional carbon efficiency?. J. Environ. Manag..

[8] Rapih S. (2022). The US vs. the EU: Cross-border bank flows’ origin factor and shadow banking in emerging market economies. Appl. Econ. Lett..

[9] Li L.Q., Ma X.G., Lu Y. (2019). The production and governance structure of enclave economy: From the perspective of state spatial restructuring. Prog. Geogr..

[10] Gallagher K.P., Zarsky L. (2007). The Enclave Economy: Foreign Investment and Sustainable Development in Mexico’s Silicon Valley.

[11] Bustos-Gallardo B. (2017). The post 2008 Chilean Salmon industry: An example of an enclave economy. Geogr. J..

[12] Hanna B. (2016). Enclaves, borders, and everyday movements: Palestinian marginal mobility in East Jerusalem. Cities.

[13] Perkmann M. (2007). Construction of new territorial scales: A framework and case study of the EUREGIO cross-border region. Reg. Stud..

[14] Gu X.K., Xie B.M., Zhang Z., Guo H.D. (2019). Rural multifunction in Shanghai suburbs: Evaluation and spatial characteristics based on villages. Habitat Int..

[15] Lian L., Ye X.T. (2016). Research on enclave economy in the coordinated development of Jingjinji region. Inq. Into Econ. Issues.

[16] Wang S.B., Luo X.L. (2022). The evolution of government behaviors and urban expansion in Shanghai. Land Use Policy.

[17] Jiang F.W., Luo X.L. (2016). Analysis to the cooperative modes of industrial parks: A case study on Jiangsu Province. Urban Probl..

[18] Phelps N.A., Miao J.T., Zhang X. (2020). Polycentric urbanization as enclave urbanization: A research agenda with illustrations from the Yangtze River Delta Region (YRDR), China. Territ. Politics Gov..

[19] Kim S.K., Terry G.V., Paul J., Mia M.B. (2022). Transboundary air pollution and cross-border cooperation: Insights from marine vessel emissions regulations in Hong Kong and Shenzhen. Sust. Cities Soc..

[20] Rui A.C., Ana V., José C.F., Luis F.P., José M.N.G., Luís C.L. (2017). Accessibility and connectivity—Movement between cities, as a critical factor to achieve success on cross-border cooperation (CBC) projects. A European analysis. Sust. Cities Soc..

[21] Xin J., Gideon B., Pieter H. (2021). Africans in Guangzhou: Is the ethnic enclave model applicable in the Chinese context?. Cities.

[22] Wen X.Z. (2004). Breaking the duke economy: From development zones to enclave economy. Sci. Technol. Ind. Parks.

[23] Liu Y.J., Luo X.L., Tian D., Wang Y. (2014). Preliminary study on China’s cross- boundary new district in forming mechanisms, spatial organizations and governance models. Econ. Geogr..

[24] Zhu H., Li G. (2015). Modes and Features for Regional Cross-border Cooperation. Urban Plan. Int..

[25] Wu F. (2016). China’s emergent city-region governance: A new form of state spatial selectivity through state-orchestrated rescaling. Int. J. Urban Reg. Res..

[26] Zhang J.X. (2013). Scale rescaling of regional governance:Based on the analysis of the perspective of “national strategic regional planning”. Urban Dev. Stud..

[27] Chen W., Yang L., Zhang P. (2021). Types, obstacles, and governance paths of regional cooperation in the YRDR. City Plan. Rev..

[28] Kwon S.W., Park S.C. (2014). Metropolitan governance: How regional organizations influence interlocal land use cooperation. J. Urban Aff..

[29] Qin X.F., Li Y.R., Lu Z., Pan W. (2020). What makes better village economic development in traditional agricultural areas of China? Evidence from 338 villages. Habitat Int..

[30] Andrew C., Doloreux D. (2012). Economic Development, Social Inclusion and Urban Governance: The Case of the City-Region of Ottawa in Canada. Int. J. Urban Reg. Res..

[31] Hu J., Sun J. (2020). Research of the regional governance model of YRDR from the perspective of state-regional scale rescaling. Urban Plan. Forum.

[32] Li S.B., Li X.M., Tian C., Cong X. (2021). Exploration of the Residents’ Quality of Life Based on the Distance of Public Service Facilities: Case of Dalian. J. Urban Plan. Dev..

[33] Smith N. (1990). Uneven Development: Nature, Capital, and the Production of Space.

[34] Wang S.B., Luo X.L., Tang M., Liu J.F. (2019). Development of cross-border integration of Beijing and Shanghai based on symbiosis theory. Sci. Geogr. Sin..

[35] Zhang B.C., Ren H., Tong X. (2017). Research on the Motivation and Mode of Industrial Cooperation Park. Mod. Manag. Sci..

[36] Scholvin S. (2021). World cities and peripheral development: The interplay of gateways and subordinate places in Argentina and Ghana’s upstream oil and gas sector. Growth Change.

[37] Clément C., Xavier D.H. (2020). Two-Way Fixed Effects Estimators with Heterogeneous Treatment Effects. Am. Econ. Rev..

[38] Liu Y.J., Zhou G.L., Liu D.G., Yu H.S., Zhu L.Y., Zhang J. (2018). The interaction of population, industry and land in process of urbanization in China: A case study in Jilin Province. Chin. Geogr. Sci..

[39] Liu F., Zhang Z.X., Zhao X.L., Liu B., Wang X., Yi L., Zuo L.J., Xu J.Y., Hu S.G., Sun F.F. (2021). Urban Expansion of China from the 1970s to 2020 Based on Remote Sensing Technology. Chin. Geogr. Sci..

[40] Lee D.W., Lee D.S. (2020). Analysis of Influential Factors of Violent Crimes and Building a Spatial Cluster in South Korea. Appl. Spat. Anal. Policy.

[41] Guo J.K., Wang S.B., Wang D.D., Liu T.B. (2017). Spatial Structural Pattern and Vulnerability of China-Japan-Korea Shipping Network. Chin. Geogr. Sci..

[42] Huallacháin B.Ó., Lee D. (2014). Urban centers and networks of co-invention in American biotechnology. Ann. Reg. Sci..

[43] Liu Z.L., Gu H.Y. (2020). Evolution Characteristics of Spatial Concentration Patterns of Interprovincial Population Migration in China from 1985 to 2015. Appl. Spat. Anal. Policy.

[44] Pang M.B., Chen C., Ma L.X. (2021). Bilevel Mixed Land Use-Transportation Model Based on Urban Road Network Balance. J. Urban Plan. Dev..

[45] Chen X., Zhong R. (2017). Cross-boundary coordination plan: A new exploration on regional governance: A case study of Jiangsu Province. Urban Plan. Int..

[46] Chan R.C.K., Xian S. (2012). Assessing The Incentivesin Regional City-to-City Cooperation:A Case Study of Jiang yinJing jiang Industrial Park of Jiangsu Province in the YangtzeRiver Delta Region. Asia Pac. View Point.

[47] Zhang P., Chen W., Wu J.W., Yuan F. (2020). Research Progress and Prospects of Cooperation Zones: Cooperation Type, Cooperation Motivation, and Cooperation Effect. Trop. Geogr..

[48] Brenner N. (2004). New State Spaces: Urban Governance and the Rescaling of Statehood.

[49] Zheng Y.T., Xu W.T., Dai L.Z. (2021). Urban growth in a post-2000 central Chinese urban agglomeration: Case study of the Changzhutan region. Growth Chang..

